# Occurrence of the Persistent Antimicrobial Triclosan in Microwave Pretreated and Anaerobically Digested Municipal Sludges under Various Process Conditions

**DOI:** 10.3390/molecules25020310

**Published:** 2020-01-12

**Authors:** Gokce Kor-Bicakci, Timothy Abbott, Emine Ubay-Cokgor, Cigdem Eskicioglu

**Affiliations:** 1 UBC Bioreactor Technology Group, School of Engineering, University of British Columbia Okanagan Campus, Kelowna, BC V1V 1V7, Canada; gokce.kor@gmail.com (G.K.-B.); tim.abbott@alumni.ubc.ca (T.A.); 2 Civil Engineering Faculty, Environmental Engineering Department, Istanbul Technical University, Maslak, 34469 Istanbul, Turkey; ubay@itu.edu.tr

**Keywords:** municipal sludge, anaerobic digestion, thermal pretreatment, microwave, contaminants of emerging concern, personal care products, antimicrobial disinfectants, triclosan, ultra-high performance liquid chromatography, tandem mass spectrometry

## Abstract

Treatment of emerging contaminants, such as antimicrobials, has become a priority topic for environmental protection. As a persistent, toxic, and bioaccumulative antimicrobial, the accumulation of triclosan (TCS) in wastewater sludge is creating a potential risk to human and ecosystem health via the agricultural use of biosolids. The impact of microwave (MW) pretreatment on TCS levels in municipal sludge is unknown. This study, for the first time, evaluated how MW pretreatment (80 and 160 °C) itself and together with anaerobic digestion (AD) under various sludge retention times (SRTs: 20, 12, and 6 days) and temperatures (35 and 55 °C) can affect the levels of TCS in municipal sludge. TCS and its potential transformation products were analyzed with ultra-high-performance liquid chromatography and tandem mass spectrometry. Significantly higher TCS concentrations were detected in sludge sampled from the plant in colder compared to those in warmer temperatures. MW temperature did not have a discernible impact on TCS reduction from undigested sludge. However, AD studies indicated that compared to controls (no pretreatment), MW irradiation could make TCS more amenable to biodegradation (up to 46%), especially at the elevated pretreatment and digester temperatures. At different SRTs studied, TCS levels in the thermophilic digesters were considerably lower than that of in the mesophilic digesters.

## 1. Introduction

The land application of biosolids is reported as the most sustainable, least expensive, and environmentally accepted disposal option for wastewater sludge management [1]. Wastewater sludge must be stabilized before recycling to soil; for reducing environmental health risks, meeting the minimum biosolids quality standards, and convincing public on its benefits and safety [2]. As a commonly used sludge stabilization method, anaerobic digestion (AD) can generate stable organic residues (e.g., biosolids) from high-strength organic waste (e.g., municipal wastewater sludge) while providing economical and environment-friendly renewable energy resources [3]. However, the release of emerging contaminants (e.g., antimicrobials) into the environment via biosolids land application is creating potential ecological and human health risks. For this reason, the detection and reduction of these problematic chemicals in sludge/biosolids has become a priority matter around the world. Although there are several studies and surveys available in the literature regarding the occurrence, fate, and endocrine disruptive effects of emerging contaminants in biosolids [4,5,6]; regulations for their occurrence in biosolids have not been established yet [7,8]. The legal framework needs to be prepared and/or updated for limiting these compounds in biosolids/agricultural soils and preventing their transfer to the groundwater and then drinking water bodies.

Triclosan (TCS) is a broad-spectrum antibacterial and antifungal agent that is effective against both Gram-positive and Gram-negative bacteria as well as yeasts and molds [9]. TCS is a polychlorinated aromatic antimicrobial which is commonly used as an active ingredient in many personal care products (e.g., soaps, shower gels, shampoos, toothpastes, and cosmetics) as well as household and industrial products (e.g., detergents, plastics, carpets, textile products, toys, and furniture) [10]. Key physical and chemical properties of antimicrobial TCS are listed in Table 1.

Municipal wastewater treatment plants (WWTPs) are generally designed to control easily/moderately biodegradable carbonaceous substances, nutrients and pathogens [20]; thus, emerging contaminants such as TCS may only be partially eliminated and/or transformed into its transformation products during treatment processes [21,22]. TCS has been widely detected in high levels (>1000 ng/g dry weight [ng/g-dry]) in municipal wastewater sludge (undigested), and has been shown to accumulate in digested biosolids and biosolids-amended agricultural soils [5,23,24,25,26] due to its physico-chemical characteristics, such as high lipophilicity and poor biodegradability. Among the 231 emerging contaminants analyzed in U.S. biosolids, TCS was identified as a priority chemical in terms of both its high occurrence and high bioaccumulation potential [5]. TCS was one of the most abundant contaminants with the mean concentration of 12,600 ± 3800 ng/g-dry, within the 72 pharmaceuticals and personal care products investigated in 110 U.S. biosolids samples collected from 94 WWTPs [8]. TCS is also commonly found in treated WWTP effluent, which then becomes another source of environmental contamination when discharged to the environment. According to the U.S. Geological Survey, TCS was identified as one of the top 10 water contaminants among the 95 compounds tested in 139 streams across 30 states [27]. Furthermore, TCS is an endocrine disrupting chemical, which has been reported to bioaccumulate in wide range of aquatic and terrestrial organisms [14,28,29]. TCS has also been detected in human samples such as urine [30], blood serum [31], breast milk [32], and maternal urine and cord blood plasma [33]. Moreover, TCS is the source of different types of chlorinated derivatives/transformation products such as dioxins/furans, chlorophenols and chloroform, which are toxic and carcinogenic compounds [34,35,36].

Until now, much attention has been paid to the identification and quantification of TCS and its transformation products in aqueous matrices (e.g., wastewater, drinking water, surface water) [9,19,37]; however, very few studies have focused on their transformation in solid matrices (e.g., municipal sludge, biosolids-amended soils, sediments) [22,35,38,39,40]. This is possibly caused by the highly-complex nature of solid matrices and the difficulties encountered during their quantitative analysis [41]. For this reason, it is important to develop easy, reliable, and cost-effective analytical methods for the determination of antimicrobials in solid matrices to help reduce the potential risks of their release and adverse effects on the environment. Despite the prevalence of TCS in wastewater sludge, a few studies have explored its fate during conventional AD. According to these studies, it can be concluded that very limited (between 0–25%) TCS removal has been observed during the AD process [22,42,43,44].

As AD is the most widely used sludge digestion technology, especially for medium- and large-scale WWTPs, it is logical to focus on solutions that can enhance the removal of TCS during anaerobic sludge digestion. In this context, different sludge pretreatment technologies applied prior to AD (i.e., advanced AD) have been used for boosting sludge disintegration and improving digestion with several advantages over the conventional AD [2]. Thermal hydrolysis, which is a highly efficient and commercially available technology, has been successfully adapted to full-scale AD operations for pretreatment [45]. As one of the most studied thermal pretreatment alternative, microwave (MW) technology has received interest from the early 2000s due to its ability to speed up the rate of reaction, heat rapidly, and provide easier operation as well as to enhance bioenergy production, sludge dewaterability, and pathogen destruction when combined with an AD process [46]. Although an extensive number of studies have been conducted to investigate the effectiveness of MW pretreatment on sludge disintegration and AD operational performance parameters (such as biogas generation, solids reduction, and sludge rheology), until now, to the best of our knowledge, there has been no study reporting on how MW pretreatment affects the level of TCS in municipal waste sludge.

The main objective of this study was to investigate the occurrence and behavior of the persistent antimicrobial TCS and its potential transformation products in undigested and anaerobically digested municipal sludges, with and without MW pretreatment. In order to achieve these goals, the following tasks were applied: (i) An ultra-high performance liquid chromatography connected to triple quadrupole mass spectrometry (UHPLC-MS/MS) based analytical method was developed for the simultaneous detection and quantification of TCS and its five potential transformation products in sludge samples, and (ii) six bench-scale, semi-continuous flow anaerobic digesters were operated with municipal mixed sludge in order to determine how different MW pretreatment temperatures (80 or 160 °C), digester sludge retention times (SRTs: 20, 12, or 6 days), and digester operating temperatures (35 or 55 °C) influence the levels of selected target compounds in anaerobically digested sludge. This study also assessed how MW pretreatment itself can affect these compounds in municipal wastewater sludge by batch sludge heating experiments.

## 2. Results and Discussion

### 2.1. Environmental Occurrence of Triclosan in Municipal Sludge

**Figure 1:**
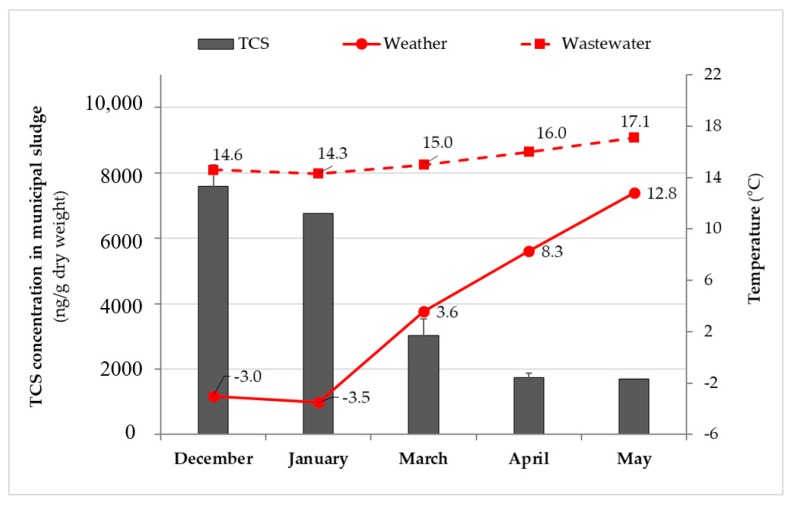
Seasonal variation in the occurrence of triclosan (TCS) in undigested mixed sludge during different sample collection periods coupled with average wastewater and weather temperatures.

The environmental (without spiking) concentration of TCS (parent compound) in municipal wastewater sludge (as a mixture of fermented primary sludge and thickened waste activated sludge (WAS)) was measured as 4450 ± 2750 ng/g-dry. However, none of the target transformation products (i.e., triclosan O-β-d-glucuronide, triclosan-*O*-Sulfate, tetra-III and penta, and 2,3,4-trichlorophenol) considered in this study could be detected in municipal sludge samples (below the instrument detection limits given in *E*-Supplementary Data). Throughout the different sampling periods, significant seasonal fluctuation in TCS concentrations of sludge samples was observed from winter to spring season (*p*-value = 0.011 < 0.05). As seen in Figure 1, TCS concentrations in the samples, which were collected from the Westside Regional WWTP during cold months (average weather and wastewater temperatures: −3 and 14 °C, respectively) of the year ranged from 6760 to 8240 ng/g-dry, whereas TCS concentrations in the samples during warmer months (average weather and wastewater temperatures: 8 and >16 °C, respectively) was in the range between 1700 and 3530 ng/g-dry. The sampling months shown in Figure 1 for undigested mixed sludge (digester feed) correspond to times when bench-scale anaerobic digesters were at the steady–state. These variations in the occurrence of TCS in sludge samples collected during the different seasonal temperatures might be related to the wastewater treatment processes applied at the facility. Specifically, the lower TCS concentrations detected in the sludge at warmer temperatures may have been caused by the higher removal of TCS (via biotransformation) in the biological nutrient removal process, utilized by the Westside Regional WWTP. As the microbial activity increased at higher temperatures compared to lower temperatures, TCS accumulation in the sludge (via sorption) may have decreased during biological process. This trend was also observed in previous studies [47,48,49]. A study by Guerra et al. [48] compared seasonal differences of TCS concentrations in WAS samples (*n* = 9 with TCS detected in 100% samples) taken from Canadian WWTPs. The TCS concentrations in the sludge were considerably higher in the samples collected in colder temperatures (4.2 ± 1.2 °C) in comparison to those collected in warmer temperatures (23.4 ± 2.4 °C). The levels of TCS were found between 630–3700 ng/g-dry in the cold season whereas between 250–910 ng/g-dry in the warm season. Tohidi and Cai [22] reported that TCS elimination efficiency in a conventional activated sludge process was significantly lower in cold sampling collection periods (34.8%) than warm periods (57%). Another study conducted by Trinh et al. [49] revealed that higher TCS removal was observed through a full-scale membrane bioreactor plant during the summer sampling period (24 ± 1 °C) compared to the winter sampling (15 ± 1 °C). Lozano et al. [38] also revealed the nitrification and denitrification processes as the most effective treatments for TCS removal (22.6%) compared to the conventional activated sludge process (10.4%).

Levels of TCS obtained from municipal sludge in this study were similar to previously reported TCS concentrations in Canadian sludge [48,50,51]. For example, TCS was found with median concentration of 4090 ng/g-dry (between 3540–4150 ng/g-dry) and 9130 ng/g-dry (between 5340–36,800 ng/g-dry) in mixed sludge samples taken from the Saskatoon and the Red Deer’s WWTPs, respectively [50]. TCS concentrations were reported from 5500 to 17,900 ng/g-dry (median: 11,550 ± 4265 ng/g-dry) in 12 municipal raw sludge collected from cities across Canada [51]. According to a study by Guerra et al. [48], the concentration ranges for TCS (*n* = 6 with TCS detected in 100% samples taken from Canadian WWTPs) were between 6000–14,000 and 430–11,000 ng/g-dry in primary sludge and thickened WAS, respectively.

### 2.2. Impact of Microwave Pretreatment on the Behavior of Triclosan in Municipal Sludge

**Figure 2:**
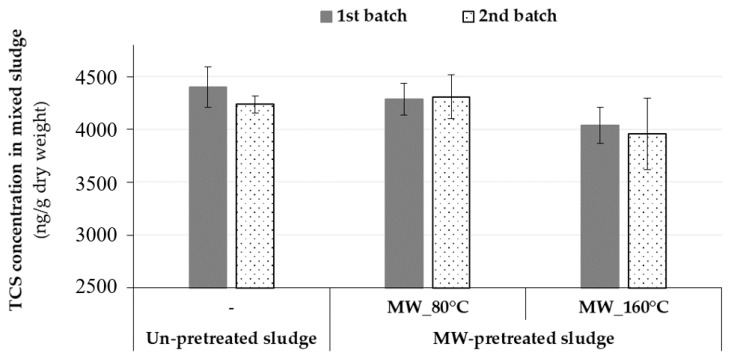
Levels of triclosan (TCS) in undigested mixed sludge before and after microwave (MW) pretreatment.

The initial studies focused on assessing MW pretreatment impact on TCS in undigested municipal sludge by applying batch heating experiments. Two independent batches of samples collected from the Westside Regional WWTP in September were analyzed in un-pretreated and MW-pretreated mixed sludges in order to evaluate the effectiveness of MW pretreatment on the reduction of TCS sludge concentrations (Figure 2). Initially, TCS was measured at an average concentration of 4320 ± 225 ng/g-dry in un-pretreated (raw) mixed sludge. Then, MW pretreatment was applied at different temperatures (80 or 160 °C); however, no discernible changes were observed in TCS sludge concentrations. After MW pretreatment, the levels of TCS in MW-pretreated mixed sludge samples were not statistically different from un-pretreated sludge according to a One-Way ANOVA (*p*-value = 0.178 > 0.05). This result might be explained by the quantum energy of MW irradiation operated at a frequency of 2450 MHz. As this activation energy (1.0 × 10^−5^ eV) is too low, MW irradiation does not provide sufficient energy to break typical chemical bonds (such as C–C: 3.61 eV, C–O: 3.74 eV, C–H: 4.28, or OH: 4.80 eV) commonly found in organic compounds [52], although it has been proven to achieve disintegration of extracellular polymeric network and pathogen destruction [46]. Based on energies of the chemical bonds, MW may not able to disrupt the specific bonds of TCS as it is a halogenated aromatic hydrocarbon. This finding was in agreement with the results of Armstrong et al. [18] who reported that TCS and some of its transformation products (methyl-TCS, and 2,4-dichlorophenol) in mixed sludge were not impacted by *Cambi Hydrolysis Process™* at temperatures of 150–180 °C for 30 min and at pressures of 0.37–0.95 MPa. In their study, the average concentrations of TCS, methyl-TCS, and 2,4-dichlorophenol before and after thermal hydrolysis ranged from 7489 to 6884 ng/g-dry, 280 to 248 ng/g-dry, and 283 to 204 ng/g-dry, respectively. On the other hand, Ross et al. [53] indicated that TCS was removed (<100 ng/g-dry) from biosolids (a mixture of WAS and anaerobically digested primary solids) when pyrolysis was performed at a temperature of 300 °C during the batch pyrolysis experiments. The authors also indicated that the pyrolysis reaction time required in order to eliminate TCS from biosolids was less than 5 min at 500 °C. Considering the results from this MW study, as well as other studies using conventional heating, it seems that higher temperatures (i.e., >300 °C, higher than TCS’s boiling point given in Table 1) are needed to observe statistically significant changes on TCS after heating.

### 2.3. Operation and Performance of Bench-Scale Conventional and Advanced Anaerobic Digesters

The semi-continuous flow anaerobic digesters achieved steady-state conditions (where solids concentration, biogas production, and pH varied by 10% or less for each digester) before data was collected for each SRT. Steady-state operations were maintained for 67, 49, and 32 days, respectively, at SRTs of 20, 12, and 6 days. All digesters were stable at each operational phase and the average organic loading rates (OLRs) of digesters were 1.46 ± 0.17, 2.57 ± 0.26, and 5.25 ± 0.42 g volatile solids (VS)/L/d, at SRTs of 20, 12, and 6 days, respectively.

**Figure 3:**
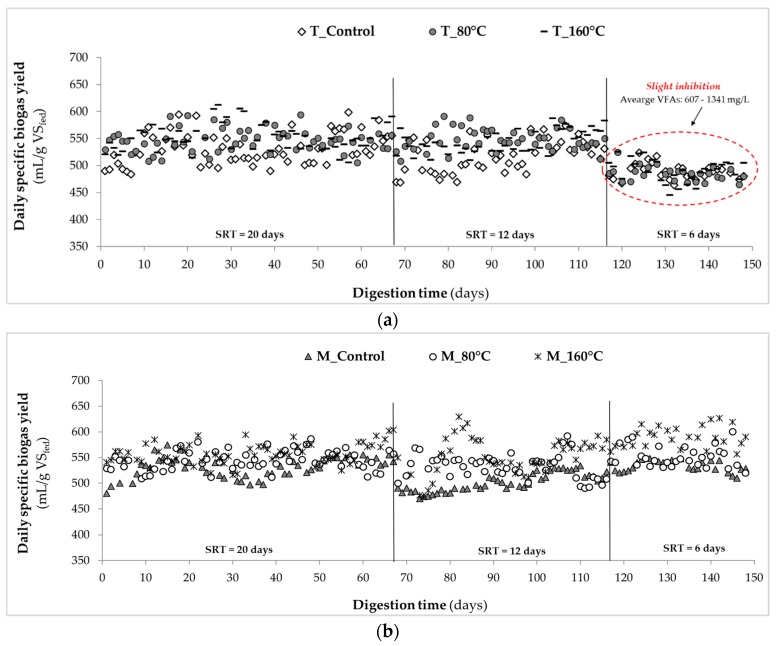
The evolution of anaerobic digesters’ specific biogas yields (0 °C, 1 atm) under (**a**) thermophilic, and (**b**) mesophilic conditions at sludge retention times (SRTs) of 20, 12, and 6 days during steady-state.

The evolution of thermophilic and mesophilic anaerobic digesters’ biogas production through the operating periods (during steady-state) is presented in Figure 3. The average daily specific biogas yields of digesters were in the range of 485–552 and 501–590 mL/g VS_fed_, respectively, under thermophilic and mesophilic temperatures. The biogas production was slightly higher in mesophilic digesters compared to thermophilic ones; however, neither operating temperature nor SRT had a significant effect on the biogas composition (*p*-value > 0.05). Throughout the AD operation, the average CH_4_ and CO_2_ contents in all digesters′ headspace were 68 ± 2 and 30 ± 2%, respectively. At SRTs of 20 and 12 days, MW-pretreated advanced digesters achieved higher biogas yields (~15%) under both operating temperatures compared to the respective controls. As the SRT was decreased to 6 days, the highest biogas generation was obtained from the advanced digester of “M_160 °C” (590 ± 23 mL/g VS_fed_; *p*-value = 0.000 < 0.05) compared to the digester of “M_Control” (532 ± 13 mL/g VS_fed_), as shown in the Figure 3b. On the other hand, a slight decrease in biogas production to 488 ± 17 mL/g VS_fed_ was observed in the thermophilic digesters at the 6-day SRT (Figure 3a), which is most likely due to the accumulation of volatile fatty acids (VFAs: up to 1341 ± 297 mg/L) under thermophilic conditions at high OLRs. Increased VFAs concentrations within the digesters can be explained by the fact that there was enough time only for the completion of hydrolysis, acidogenesis, and acetogenesis steps, but not adequate time for the completion of methanogenesis. The inhibition behavior observed from thermophilic systems are in agreement with literature reporting on reduced microbial diversity and therefore less stable AD operation under high OLRs and/or when utilizing by-products of high intensity pretreatment at elevated digester temperatures [54,55].

**Figure 4:**
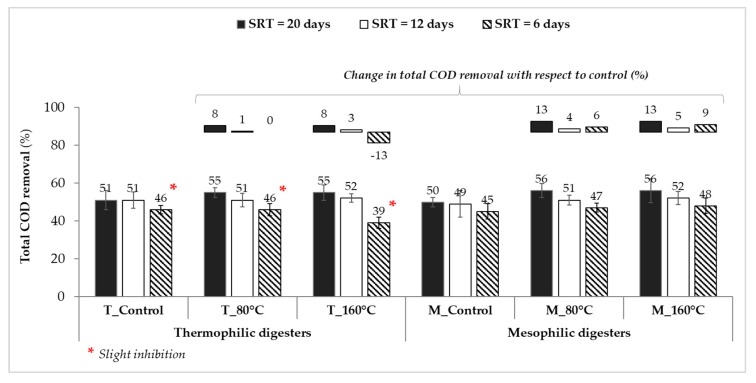
Average total chemical oxygen demand (COD) removal efficiencies of thermophilic and mesophilic anaerobic digesters at SRTs of 20, 12, and 6 days.

Organic matter destruction efficiency across the digester is used as one of the key operational parameters while determining the performance of digesters. During the operation of three SRTs, the average removal efficiencies of total chemical oxygen demand (COD) varied between 39%–55% and 45%–56% in the thermophilic and mesophilic digesters, respectively (Figure 4). As expected, higher total COD removal efficiencies (up to 56%) were achieved at longer SRTs under both operating temperatures. Advanced digesters that were fed with MW-pretreated sludge accomplished higher total COD removals compared to the respective controls, except for the thermophilic digesters operated at the shortest SRT (due to mild VFA inhibition). Specifically, the digester of “T_160 °C” had 13% reduction in total COD removal efficiency compared to the respective control (Figure 4). Based on the improvement results in biogas production and organics removal, it can be concluded that advanced AD coupled with MW pretreatment was able to tolerate ~15% higher loading rates compared to the control digesters for the lowest SRT.

Other conventional parameters such as pH, alkalinity, and ammonia were also analyzed during each operational phase to monitor each digester’s performance. The daily pH values of digesters were stable between 7.7 and 8.1 under thermophilic temperature and between 7.3 and 7.5 under mesophilic temperature. Average alkalinity concentrations of the thermophilic and mesophilic digesters were in the range of 3900–5600 and 3000–4400 mg/L as CaCO_3_, respectively. Average ammonia–nitrogen concentrations ranged from 1150 to 1700 and from 800 to 1400 mg N/L for thermophilic and mesophilic digesters, respectively. These results also confirmed stable operation of all digesters during different SRTs without process failure.

### 2.4. Triclosan Occurrence after Conventional and Advanced Anaerobic Sludge Digestion

**Figure 5:**
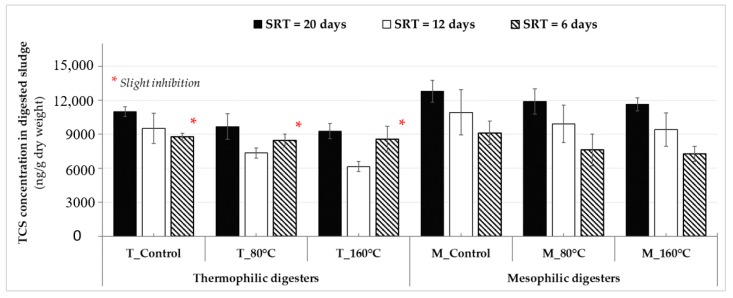
Average triclosan (TCS) concentrations in anaerobically digested sludge obtained from thermophilic and mesophilic digesters at sludge retention times (SRTs) of 20, 12, and 6 days during steady-state.

Average concentrations of TCS in digested sludge taken from conventional (without pretreatment) and advanced anaerobic digesters that utilized MW irradiation at steady-state under thermophilic and mesophilic conditions are presented in Figure 5. At different SRTs, the levels of TCS in thermophilic digestates were lower than that of in mesophilic digestates (*p*-value = 0.01 < 0.05 for the 20-day and *p*-value = 0.03 < 0.05 for the 12-day). For instance, the digester of “T_Control” had an average TCS concentration of 10,990 ± 440 ng/g-dry in its effluent at an SRT of 20 days whereas the digester of “M_Control” had 12,790 ± 965 ng/g-dry (*p*-value = 0.057 > 0.05). This indicates that the long SRT combined with the higher digester temperature (20-day/55 °C) in “T_Control” created more favorable conditions for TCS reduction compared to “M_Control”. As a combined effect (MW pretreatment coupled with AD), the use of MW pretreatment prior to AD process contributed to some extent on the reduction of TCS levels in digested sludge compared to conventional AD at each operational phase. At the 20-day SRT, the average TCS concentrations decreased to 9680 ± 1130 and 9280 ± 660 ng/g-dry in the thermophilic MW-pretreated digesters of “T_80 °C” and “T_160 °C”, respectively (Figure 5). The lowest TCS concentration (6145 ± 450 ng/g-dry) was achieved from the digester of “T_160 °C” fed with pretreated sludge at 160 °C, under thermophilic temperature at the 12-day SRT. At the final 6-day SRT, no significant differences were observed between the average TCS concentrations in digested sludge samples from conventional and MW-pretreated digesters operated at thermophilic temperature (*p*-value = 0.925 > 0.05). This could be due to the reduced biological treatment process performance observed from the advanced digesters pretreated at high temperatures at the shortest SRT. Similar to the aforementioned AD performance results that reported slight inhibition caused by VFAs accumulation (in Section 2.3), mesophilic advanced digesters operated at the 6-day SRT also had better performance on the elimination of TCS from digested sludge compared to thermophilic advanced digesters (Figure 5). It is also worthy to note that although the effect of MW irradiation itself was not statistically significant on TCS concentration of mixed sludge (Figure 2), when the effect was combined with effect of AD (SRT and temperatures), the advanced digesters were more successful in TCS reduction compared to their control counterparts (Figure 5). This suggest that although MW cannot break apart the bonds of TCS during pretreatment at the temperature range studied (80 and 160 °C), it can make micropollutants (originally part of the polymeric network) more amenable (accessible) to biological degradation by disintegrating/solubilizing extracellular polymeric network and releasing them into soluble phase. This behavior was also reported for steroidal hormones in MW pretreated digesters utilizing municipal sludge cake [56].

The concentrations of TCS measured in anaerobically digested sludge in this study were similar to results of earlier studies [50,57,58]. According to study by Guerra et al. [57], TCS was found in all biosolids samples (*n* = 24) collected from six WWTPs at levels from 2000 to 11,000 ng/g-dry (median: 6800 ng/g-dry). TCS was measured (with 97% occurrence) in treated sludge and biosolids (*n* = 31) taken from the different regions of Canada with the median concentration of 6085 ng/g-dry [50]. The concentration levels of TCS in anaerobically digested biosolids collected from mesophilic digesters in the Red Deer’s WWTP were between 11,700 and 13,900 ng/g-dry, whereas TCS concentrations were between 5590 and 6270 ng/g-dry in the Saskatoon WWTP, located in Canada [50]. TCS concentrations in treated biosolids obtained from four WWTPs in Ontario were also reported as in the range of 680–11,550 ng/g-dry [58].

Although high levels of TCS were detected in digestate samples in this study, its transformation products monitored were below the instrument detection limits (*E*-Supplementary Data) in sludge samples. These results highlight the need for further investigation on the formation of TCS’s other transformation products (such as methyl-TCS or 2,4-dichlorophenol) during AD.

When it comes to the effect of digester SRT, the levels of TCS in the thermophilic and mesophilic anaerobic digesters’ effluents (except for the thermophilic MW-pretreated digesters at the 6-day SRT) decreased as the SRT was reduced (Figure 5), which was unexpected. Decreasing TCS concentrations in digested sludge may have been caused by the seasonal temperature variation, which followed a similar trend with the fluctuation of TCS levels monitored in the municipal sludge at the different sampling periods (in Section 2.1). In this study, the waste sludge samples used for digester feeding were collected from the WWTP between the months of “September–December”, “January–March”, and “March–April” during SRTs of 20, 12, and 6 days, respectively. For this reason, the concentrations of TCS in digestates might be higher at colder (winter) temperatures compared to that of at warmer (spring) temperatures due to higher levels of TCS entering AD systems. These results are consistent with the findings of the previous study conducted by Guerra et al. [48]. The authors found higher levels of TCS in aerobically digested biosolids collected in colder temperatures (190–1600 ng/g-dry) compared to those collected in warmer temperatures (69–930 ng/g-dry). Making the comparison based on rate of TCS mass loading represented as ng/d (calculated from Equation (1), *Materials and Methods* section), rather than concentration (ng/g-dry) facilitates comparison among various SRTs.

**Figure 6:**
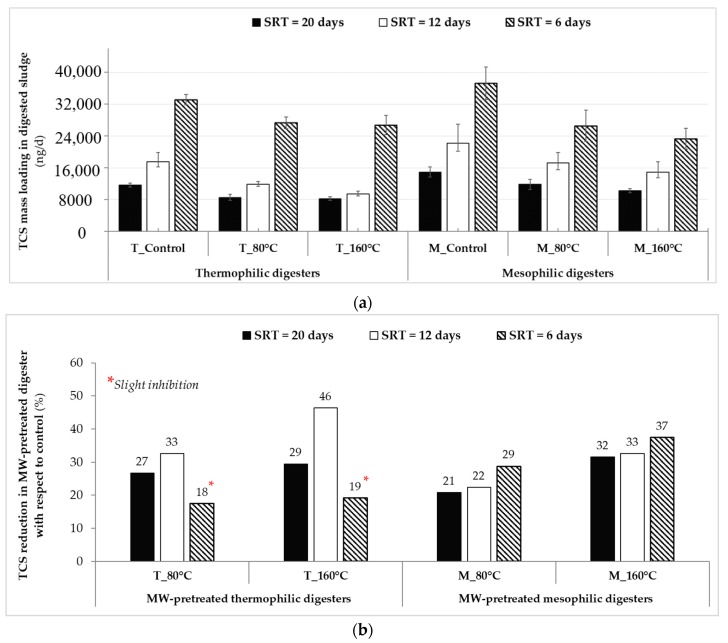
(**a**) Daily triclosan (TCS) mass loadings in anaerobically digested sludge obtained from thermophilic and mesophilic digesters at sludge retention times (SRTs) of 20, 12, and 6 days during steady-state, and (**b**) the reduction percentage of TCS mass loadings exiting from MW-pretreated digesters compared to their respective control digesters.

The rate of mass loading determined for TCS in the conventional and advanced AD systems at different SRTs is represented in Figure 6a. The daily average TCS mass loadings in digested sludge samples were between 8230–14,935 ng/d at the 20-day SRT, between 9385–22,145 ng/d at the 12-day SRT, and between 23,295–37,230 ng/d at the 6-day SRT. When it comes to understanding the mechanisms of changes occurred in TCS levels during AD, photodegradation and volatilization can be assumed negligible, because the nature of TCS is relatively non-volatile. This observation could only be explained by the microbial activity during AD, which may have resulted in TCS degradation via biotransformation and/or mineralization.

Similar to the trend reported in Figure 5, the mass loading comparison also confirmed that TCS removal from digested sludge was increased in the advanced digesters compared to conventional digesters (Figure 6a). The reduction percentage of TCS mass loading exiting from MW-pretreated digesters compared to the respective control digesters (calculated from Equation (2) in *Materials and Methods* section) is shown in Figure 6b. Digesters utilizing low-temperature microwaved sludge (80 °C) improved TCS reduction in digested sludge by 18%–33%, and 21%–29%, respectively under thermophilic and mesophilic operating temperatures at different SRTs, compared to controls. The levels of TCS reduction in the advanced digesters operated at thermophilic temperatures (except at the 6-day SRT) were higher compared to that of the digesters at mesophilic temperatures. Under thermophilic conditions, the lowest rate of TCS mass exiting from the digester utilizing microwaved sludge at 160 °C was 9385 ± 745 ng/d at the 12-day SRT, and resulted in the highest TCS reduction (46%) compared to the respective control (17,500 ± 2310 ng/d). However, the reduction of TCS from the mesophilic digester of “M_160 °C” accounted only for 33% compared to the respective control (Figure 6b). On the other hand, the same mesophilic digester displayed moderate reduction of TCS (37%) over the respective control at the 6-day SRT. In general, these results confirm that compared to controls, MW irradiation makes TCS more available to microbial assisted transformation within AD and the effect is most pronounced at the highest MW temperatures (160 °C) combined with thermophilic AD under an OLR of 2.57 ± 0.26 g VS/L/d.

Initially it was expected that improvements (%) in TCS removals in hybrid AD systems (MW + AD) compared to controls (AD) would increase as SRT was reduced from 20 days to 12 and 6 days as controls were expected to be challenged at shorter SRTs. However, in this study, when the SRT was reduced, TCS reduction obtained from the digesters increased at both mesophilic and thermophilic (except for “T_80 °C” and “T_160 °C” that experienced slight inhibition at the 6-day SRT) temperatures (Figure 6b). Aside from the MW irradiation affect, this can be explained by the higher accessibility of micropollutants in digested sludge matrix compared to digester feed sludge during extraction step of analytical analysis. In other words, micropollutants can be more accessible to quantification in digested sludge compared to that of in undigested sludge due to the alteration of organic matter quality and quantity during AD process (both in control and pretreated ADs). Consequently, as the extent (i.e., SRT) of digestion increased, concentrations of micropollutants (such as TCS levels in this study) in effluents from AD would increase due to higher detectability/quantification compared to that in effluents at shorter SRTs (i.e., 12 or 6 days), and resulting in reduced micropollutant reduction percentages for long SRTs, as previously reported for pharmaceuticals in sludge digesters [59].

### 2.5. Feasibility of Technology at Full-Scale

Although the results from this study indicate that MW pretreatment was effective in decreasing TCS levels in anaerobically digested sludge when combined with AD, microwave systems at 2450 MHz would not be feasible for full-scale implementation because of inefficient heating of wastewater sludge leading to higher electricity requirements [54]. However, recent studies [60] indicate that optimizing heating frequency and pretreatment equipment design for sludge dielectric properties will increase net energy production significantly at full-scale, while retaining process benefits of sludge pretreatment in terms of digester operation and digestate quality.

## 3. Materials and Method

### 3.1. Municipal Wastewater Sludge

Fermented primary sludge and thickened WAS were taken every two weeks from the Westside Regional WWTP (West Kelowna, B.C., Canada) which has a capacity of 16,800 m^3^/day. This facility employs preliminary, and primary treatment processes followed by secondary (biological nutrient removal through a three-phase modified Bardenpho process) and tertiary (filtration and UV disinfection) treatments.

### 3.2. Experimental Methodology

MW pretreatment of thickened WAS samples was carried out in a *Milestone 2.45 GHz MW Lab Station* (ETHOS-EZ: maximum pressure of 35 bar, maximum temperature of 300 °C, and maximum power of 1200 W). At a constant ramp rate of 2.25 °C/min, thickened WAS samples were irradiated to desired temperatures of 80 and 160 °C which represented the low- and high-temperature pretreatment conditions as below and above boiling point temperatures, respectively. Samples were held at these temperatures for 30 min, and then cooled until ambient room temperature. Microwaved thickened WAS samples were mixed with raw fermented primary sludge with a volume % ratio of 67:33, respectively, to provide MW-pretreated mixed sludge samples. Three different mixed sludge were prepared for feeding the digesters: one un-pretreated and two pretreated samples. The basic characteristics of the different sludge streams and mixed sludge samples are shown in Table 2.

Six side-armed Erlenmeyer flasks were used to construct bench-scale anaerobic digesters (fed once a day, 7 days/week). Total and liquid volumes of the digesters were 2 and 1 L, respectively. The start-up of the digesters was done by taking mesophilic and thermophilic inocula from existing bench-scale digesters which had been operating using similar mixed sludge for over one year. Digesters were operated at SRTs of 20, 12, and 6 days under thermophilic (55 ± 1 °C) and mesophilic (35 ± 1 °C) temperatures. The summary of experimental design used for MW pretreatment and anaerobic digesters is provided in Table 3. Further information about the experimental methodology of this study can be found in the previous publication of the authors [54].

### 3.3. Analytical Methodology

Once steady-state conditions were reached during each operational phase (after a period corresponding to minimum three SRTs), anaerobically digested sludge samples (*n* = 3 or 4) were taken from the digesters’ effluent streams for the simultaneous detection and quantification of target compounds (analytes: parent compound [*TCS*] and its five transformation products [*triclosan O-β-d-glucuronide, triclosan-O-Sulfate*, chlorophenoxyphenol derivatives (*tetra-III* and *penta*), and chlorophenol derivative (*2,3,4-trichlorophenol*)]), and characterization of digesters performance via conventional parameters. For quantification of the target compounds in control digester feed (mixed sludge), a total of 15 independent undigested mixed sludge samples were analyzed (between the months of December to May). For sample preparation, after sampling, thickened WAS and fermented primary sludge samples were first mixed in the laboratory at a volume % ratio of 67:33, respectively (based on the plant’s operation), then the mixed sample was processed for TCS quantification (Figure 1). Additionally, two independent batches of samples were taken from the plant during September to assess the effectiveness of MW pretreatment itself on these target compounds’ concentrations. MW treated mixed sludge samples (4 samples in total) were prepared by mixing pretreated thickened WAS and raw fermented primary sludge samples at a volume % ratio of 67:33. Then TCS quantification was done for the pretreated mixed sludge samples along with the unpretreated mixed sludge (prepared the same way described above) (Figure 2). Although other TCS transformation products have been detected in literature (i.e., methyl-triclosan, 2,8-dichlorodibenzo-*p*-dioxin), the compounds selected for this study were based on the availability of native standards and their suitability for UHPLC analysis.

#### 3.3.1. Analysis of Triclosan and Its Transformation Products

##### Chemicals

TCS (CAS# 3380-34-5; 99.7 ± 0.2% purity) was acquired by Sigma Aldrich (Oakville, ON, Canada). TCS transformation products including triclosan O-β-d-glucuronide (CAS# 63156-12-7), and triclosan-*O*-Sulfate (CAS# 68508-18-9) were purchased from Toronto Research Chemicals (Toronto, ON, Canada) while tetra-III (CAS# 63709-57-9) and penta (CAS# 53555-01-4) were obtained from Wellington Laboratories (Guelph, ON, Canada). 2,3,4-trichlorophenol (CAS# 15950-66-0, ≥98% purity) was acquired through Sigma Aldrich (Oakville, ON, Canada). Isotopically labelled triclocarban-d_4_ (TCC-d_4_, CAS# 1219799-29-7, >99% atom deuterated) was purchased from C/D/N Isotopes Inc. (Pointe-Claire, QC, Canada). Additional information about target analytes are given in Table S1 (*E*-Supplementary Data in online version). Stock solutions of native compounds were prepared in UHPLC grade methanol (A456, Fisher Scientific, Ottawa, ON, Canada).

##### Sample Preparation and Extraction

**Figure 7:**
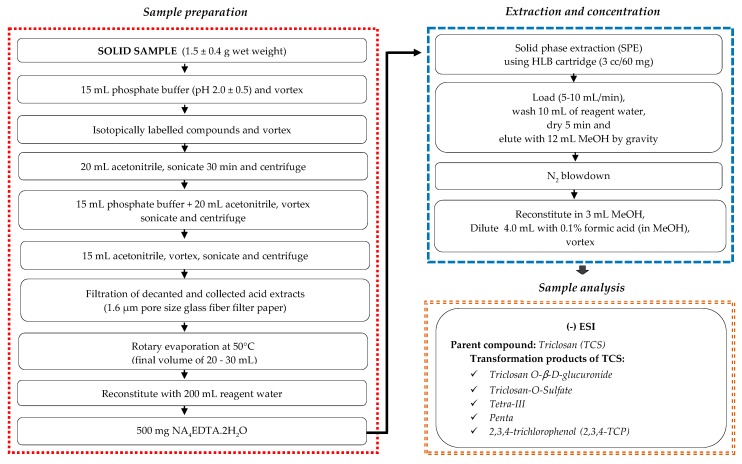
Flow chart for simultaneous detection and quantification of triclosan and its transformation products in sludge (adapted from *U.S. EPA Method 1694* [61]).

Sludge samples were prepared and extracted according to the acid fraction procedure of the *U.S. EPA Method 1694* with some modifications [61]. A flow chart that summarizes procedures for sample preparation, cleanup, concentration, and analysis steps of target analytes in the sludge samples, is given in Figure 7. Further detailed information regarding each step of procedure is provided in Kor-Bicakci [62] and Kor-Bicakci et al. [63].

##### Instrumental Analysis

A Waters™ ACQUITY UHPLC coupled with a Waters™ Xevo TQD triple quadrupole mass spectrometer (MS) equipped with an electrospray ionization (ESI) probe was utilized for the simultaneous detection and quantification of TCS and its transformation products in sludge samples. Various methods for the extraction and detection of TCS and several TCS metabolites have been reported in literature. U.S. EPA 1694 served as the basis for sample extraction and provided a starting point for UHPLC and MS/MS methods. Numerous trials of various aqueous phase additives, UHPLC gradients, mobile phase modifiers, injection volumes, MS conditions etc. were performed to optimize the chromatography (peak shape and compound separation) and to maximize the sensitivity of the MS, while minimizing noise. However, environmental levels of TCS (non-spiked) and the limited biodegradability of TCS during AD resulted in even lower TCS metabolite levels that were challenging to quantify. Furthermore, the low mass to charge ratio (*m*/*z*) of TCS fragment ions as well as the fragment ions of several TCS metabolites made ultra-low detection and quantification limits difficult in such a complex sludge matrix.

After multiple trials, the following analysis conditions were selected. The UHPLC was run using an aqueous mobile phase of 5 mM of UHPLC grade ammonium acetate (AX1222-5, VWR, Mississauga, ON, Canada) solution and an organic phase of UHPLC grade methanol. Analytes were separated using a Waters™ Ethylene Bridged Hybrid C18 (2.1 × 50 mm, 1.7 μm) column and matching guard column. MS acquisition was performed in multiple reaction monitoring (MRM) using the negative ion mode (ESI-) for each analyte. Retention times of analytes were within ±15 s of a native compound as per U.S. EPA Method 1694 [61]. Further details regarding optimized MS/MS conditions used for each analyte quantification are provided in the Supplemental Information in Table S2 (*E*-Supplementary File).

##### Method Validation

A multi-point calibration curve was prepared from native compounds to quantify each analyte (minimum of 8 and maximum of 12 calibration points). Quantification was performed by an isotope dilution technique using isotopically labelled TCC-d_4_ as an internal standard. Good linearity was yielded with a correlation coefficients (R^2^) ≥ 0.98 for all analytes. The instrument limit of detection (LOD) and limit of quantification (LOQ) were calculated using the signal-to-noise ratio of 3 and 10 or greater in native standards or 9 in samples, respectively. Recoveries for most analytes were within the EPA recommended range of 70%–130% with some exceptions [61]. Compound-specific LODs, LOQs, reporting limits, and recoveries are provided in Table S3 (*E*-Supplementary Data).

##### Mass Loading Calculation for Triclosan

The daily rate of TCS mass exiting from the conventional and advanced AD system (as anaerobically digested sludge) was calculated with Equation (1):

(1) $$M_{eff}{\;=\;Q}_{eff}\;\times {\;TS}_{eff}\;\times {\;C}_{eff}$$

*M_eff_* = Daily TCS mass loading in the effluent of the digester (ng/d),
*Q_eff_* = Flow rate of anaerobically digested sludge (digestate) (mL/d),
*TS_eff_* = Total solids concentration of anaerobically digested sludge (% by weight), and
*C_eff_* = TCS concentration in anaerobically digested sludge (ng/g-dry).

The reduction percentage of TCS mass loading exiting from MW-pretreated digesters compared to the respective control digesters was then calculated from Equation (2):

(2) $$TCS\;reduction\;(\%)\;=\;\frac{M_{control\;digester}{\;-\;M}_{microwave\;pretreated\;digester}}{M_{control\;digester}}\;\times \;100$$

*M_control digester_* = Daily TCS mass loading in the effluent of the control digester (ng/d), and
*M_microwave pretreated digester_* = Daily TCS mass loading in the effluent of MW-pretreated digester (ng/d).

#### 3.3.2. Conventional Parameters

Total solids, VS, COD, pH, alkalinity, and ammonia were analyzed according to Standard Methods [64] procedures 2540 B, 2540 E, 5250 D, 4500-H^+^B, 2320 B, and 4500-NH_3_D, respectively. Digester biogas was collected in Tedlar^®^ bags and was measured daily by a U-Tube type manometer, while its composition was analyzed by an Agilent 7820A Gas Chromatograph equipped with a thermal conductivity detector [65]. Total VFAs (sum of acetic, propionic and butyric acids) were measured by an Agilent 7890A Gas Chromatograph with a flame ionization detector and Agilent 19091F-112 capillary column [66].

### 3.4. Statistical Analysis

The experimental data were analyzed with an analysis of variance (ANOVA) considering a 95% confidence interval (α = 0.05), using Minitab™ 17 statistical software.

## 4. Conclusions

This study assessed the behavior of the antimicrobial TCS in MW-pretreated and anaerobically digested municipal sludges under various process conditions by batch MW pretreatment and semi-continuous flow AD experiments, respectively. The combined effect of AD coupled with MW pretreatment on the occurrence of TCS in wastewater sludge was studied for the first time. All digesters maintained steady-state operation during each of the three SRTs studied. Slight inhibition was observed in the thermophilic digesters operated at the 6-day SRT; nevertheless, these digesters continued producing daily biogas with a typical methane content of 65%. Initial batch pretreatment studies indicated that TCS levels in mixed sludge after MW pretreatment applied at 80 or 160 °C did not significantly change in comparison to un-pretreated sludge. Compared to conventional AD, the combination of anaerobic sludge digestion with MW pretreatment moderately favored the reduction of TCS levels in digested sludge. The advanced thermophilic digesters achieved up to 46% TCS reduction from digested sludge over the respective controls. Compared to the thermophilic temperature, the mesophilic digester temperature was less effective on decreasing TCS levels in digested sludge (up to 37%). Higher TCS concentrations were observed in undigested and anaerobically digested municipal sludges during colder sludge sampling periods. This study confirmed the impacts of seasonal temperature variations on the levels of TCS in municipal wastewater sludge. Although TCS concentrations varied seasonally, MW pretreatment was effective in decreasing TCS levels in anaerobically digested sludge when combined with AD.

## Figures and Tables

**Table 1:** Key physical and chemical properties of triclosan.

Parameters	Triclosan (TCS)
Chemical structure	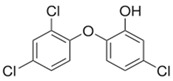
Molecular formula	C_12_H_7_Cl_3_O_2_
Molecular weight (g/mol)	289.536
CAS registration number	3380-34-5
IUPAC name	2,4,4′-trichloro-2′-hydroxydiphenyl ether or 5-chloro-2-(2,4-dichlorophenoxy)phenol
Trade name	Irgasan DP300 or CH 3565
Use	Antiseptic and disinfectant
Chlorine content (weight %)	33.7 ^a^
Log *K*_ow_ (at 25 °C, pH 7)	4.8 ^a^
Log *K*_oc_ (at 25 °C, pH 7)	4.1 ^b^
p*K*_a_ (at 20 °C)	8.14 ^c^
Melting point (°C)	54 – 57 ^c^
Boiling point (°C)	280–290 ^d^
Water solubility (mg/L at 25 °C)	1.97–4.6 ^a^
Vapour pressure (mm Hg at 25 °C)	4.65 × 10^−6 e^
Some of potential transformation products ^f,g h,i,j^	Methyl-triclosan 2,4-dichlorophenol 2,4,6-trichlorophenol 2,3,4-trichlorophenol 2,8-dichlorodibenzo-p-dioxin Triclosan O-β-d-glucuronide Triclosan-O-Sulfate Tetra-III Penta Chloroform

Log *K*_ow_: The logarithmic octanol-water partition coefficient, Log *K*_oc_: Organic carbon partition coefficient, p*K*_a_: Dissociation constant. ^a^ Halden and Paull [11], ^b^ Heidler and Halden [12], ^c^ Reiss et al. [13], ^d^ Dann and Hontela [14], ^e^ Ying et al. [15], ^f^ Tohidi and Cai [16], ^g^ Chen et al. [17], ^h^ Armstrong et al. [18], ^i^ Dann and Hontela [14], ^j^ Canosa et al. [19].

**Table 2:** Basic characteristics of waste sludge streams and mixed sludge samples.

Waste Sludge Sample			Parameter			
			pH (-)	TS (% *w*/*w*)	VS (% *w*/*w*)	VS/TS (%)
Thickened waste activated sludge			6.15 (0.20; 5) ^a^	3.93 (0.48; 12)	3.18 (0.40; 12)	80.9
Fermented primary sludge			5.27 (0.15; 5)	7.10 (0.96; 12)	6.47 (0.89; 12)	91.1
Mixed sludge	*Un-pretreated*		5.63 (0.13; 5)	3.94 (0.24; 16)	3.38 (0.20; 16)	86.0
	*Pretreated*	MW_80 °C	5.64 (0.05; 5)	3.40 (0.35; 16)	2.90 (0.33; 16)	85.3
		MW_160 °C	5.52 (0.20; 5)	3.37 (0.25; 16)	2.87 (0.24; 16)	85.2

TS: total solids, VS: volatile solids, MW: microwave. ^a^ Data displays the arithmetic mean of measurements (standard deviation; number of data points).

**Table 3:** Summary of the experimental methodology used.

*Microwave Pretreatment*		Mixed Sludge Sample Name	*Anaerobic Digestion*		
Temperature ^a^ (°C)	Exposure Duration (min)		Digester Name	Digester Type	SRT (days)
-	-	Un-pretreated	T_Control M_Control	Thermophilic Mesophilic	20, 12, and 6
80	30	MW_80 °C	T_80 °C M_80 °C	Thermophilic Mesophilic	20, 12, and 6
160	30	MW_160 °C	T_160 °C M_160 °C	Thermophilic Mesophilic	20, 12, and 6

SRT: sludge retention time, T: thermophilic, M: mesophilic, MW: microwave. ^a^ Samples were pretreated at a constant heating ramp rate of 2.25 °C/min.

## Supplementary Materials

The following are available online, Table S1: Further information about triclosan and five of its transformation products, Table S2: Optimized MS/MS conditions used for each analyte quantification, and Table S3: Validation of developed method for each analyte.
