# Supplementary material for: Occurrence of the Persistent Antimicrobial Triclosan in Microwave Pretreated and Anaerobically Digested Municipal Sludges under Various Process Conditions
Source: Molecules. 2020 Jan 12;25(2):310. doi: 10.3390/molecules25020310 (PMC7024389; doi:10.3390/molecules25020310)
Supplement: Supplementary file 1 [file molecules-25-00310-s001.pdf]

Supplementary Materials for

Occurrence of the persistent antimicrobial triclosan in microwave pretreated and anaerobically digested municipal sludges under various process conditions

Gokce Kor-Bicakci [1,2], Timothy Abbott [1], Emine Ubay-Cokgor [2] and Cigdem Eskicioglu [1,*]

[1] UBC Bioreactor Technology Group, School of Engineering, University of British Columbia Okanagan Campus, Kelowna, British Columbia V1V 1V7, Canada; gokce.kor@gmail.com (G.K.B.); tim.abbott@alumni.ubc.ca (T.A.)

[2] Istanbul Technical University, Civil Engineering Faculty, Environmental Engineering Department, 34469 Maslak, Istanbul, Turkey; ubay@itu.edu.tr (E.U.C.)

* Correspondence: cigdem.eskicioglu@ubc.ca (C.E.); Tel.: +1-250-807-8544.

Tables	Page
**Table S1.** Further information about triclosan and five of its transformation products ……..	2
**Table S2.** Optimized MS/MS conditions used for each analyte quantification ………………..	3
**Table S3.** Validation of developed method for each analyte …………………………….......	4

Table S1. Further information about triclosan and five of its transformation products.

Analyte	IUPAC Name	Molecular Weight (g/mol)	Chemical structure
Parent compound			
Triclosan (TCS)	5-chloro-2-(2,4-dichlorophenoxy)phenol	289.54	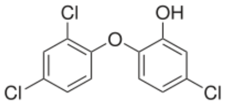
Transformation products			
Triclosan O-β-D-glucuronide	5-chloro-2-(2,4-dichlorophenoxy)phenyl β-D-glucopyranosiduronic acid	487.65[a]	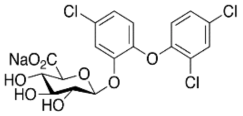
Triclosan-*O*-Sulfate	Sodium 5-chloro-2-(2,4-dichlorophenoxy)phenyl sulfate	391.59[a]	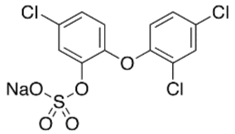
Tetra-III	5,6-dichloro-2-(2,4-dichlorophenoxy)phenol	323.978	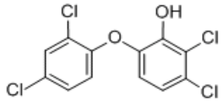
Penta	4,5,6-trichloro-2-(2,4-dichlorophenoxy)phenol	358.432	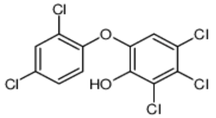
2,3,4-TCP	2,3,4-trichlorophenol	197.45	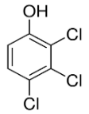
Internal standard			
Triclocarban (TCC-d_4)	1-(4-chlorophenyl)-3-(3,4-dichlorophenyl)urea-d_4	319.61	-

[a]as sodium salt.

Table S2. Optimized MS/MS conditions used for each analyte quantification.

Compound	Ion Source	Parent ion (m/z)	Cone potential (V)	Fragment ion (m/z)	Collision Energy (V)	Capillary voltage (kV)	
						[a]*ESI-*	[b]*ESI+*
Parent compound:							
Triclosan (TCS)	ESI-	286.9	20	34.9	10	0.6	0.6
Transformation products:							
Triclosan O-β-D-glucuronide	ESI-	465.0	34	113.0	18	0.6	0.6
Triclosan-*O*-Sulfate	ESI-	368.9	34	288.9	18	0.6	0.6
Tetra-III	ESI-	322.8	24	34.9	12	0.6	0.6
Penta	ESI-	356.8	26	34.8	14	0.6	0.6
2,3,4-trichlorophenol	ESI-	194.8	38	158.8	18	0.6	0.6
Triclocarban-d_4 (TCC-d_4)	ESI-	317.0	44	129.9834<br>159.9541	24, 14	varies	varies

[a]negative ionization mode, [b]positive ionization mode.

Table S3. Validation of developed method for each analyte.

Compound	LOD[a] (ppb)	LOQ[b] (ppb)	RL[c] (ppb)	Recovery (%)
Parent compound:				
Triclosan (TCS)	0.33	0.50	1.10	112.50
Transformation products:				
Triclosan O-β-D-glucuronide	1.44	5.22	5.46	107.24
Triclosan-*O*-Sulfate	0.12	0.31	0.50	317.64
Tetra-III	0.58	0.95	4.92	110.3
Penta	0.09	0.25	0.46	105.18
2,3,4-trichlorophenol	0.26	0.57	0.69	114.2

[a]LOD: limit of detection, [b]LOQ: limit of quantification, [c]RL: reporting limit.
